# Major intraoperative bleeding and drastic change in circulatory dynamics in a pregnant patient with metastatic pheochromocytoma: a case report

**DOI:** 10.1186/s40981-022-00504-9

**Published:** 2022-02-22

**Authors:** Sumire Kurosawa, Hiroyuki Yamasaki, Wakuya Hasegawa, Takashi Mori

**Affiliations:** Department of Anesthesiology, Osaka City University Graduate School of Medicine, 1-5-7 Asahimachi, Abeno-ku, Osaka City, Osaka, 545-8586 Japan

**Keywords:** Metastatic pheochromocytoma, Extra-adrenal pheochromocytoma, Spinal tumor, Pregnant patient, Prone position

## Abstract

**Background:**

Metastatic pheochromocytoma in the spine is a very rare complication during pregnancy. We report anesthesia in a pregnant woman for resection of an undiagnosed spinal tumor, accompanied by remarkable hemodynamic changes and massive bleeding.

**Case presentation:**

A 33-year-old woman at 17 weeks of gestation presented with the rapid progress of bilateral lower leg paralysis. A diagnosis of spinal tumor was made, and surgical resection was planned. Although the surgery was suspended because of remarkable hemodynamic changes and massive bleeding, fetal heart rate was stable. Postoperative examination revealed pheochromocytoma in the urinary bladder as a primary lesion with spinal metastasis.

**Conclusion:**

Although spinal pheochromocytoma is extremely rare in pregnant women, it should be suspected when abnormal hypertension is observed with accompanying neurological deficits. Preservation of maternal circulation and uteroplacental blood flow should be the first priority during anesthesia.

## Background

Pheochromocytoma is an extraordinarily rare complication, with a frequency of 0.002% in all pregnancies [1]. The diagnosis of pheochromocytoma is often difficult in pregnant women because of its extreme rarity and similar symptoms commonly observed with pregnant women, particularly at the early stage of pregnancy. Metastatic forms account for nearly 10% of all cases of pheochromocytoma [2]; however, metastatic spread to the spine is very rare [3]. Spinal metastasis may cause rapid progress of neurological deficits and require emergency surgery; however, diagnostic use of CT or nuclear imaging should be avoided due to the radiation risk during pregnancy, which makes a definitive diagnosis to be more difficult [4, 5]. We report a pregnant woman with a spinal tumor manifested with rapidly progressive bilateral paralysis of the lower extremities, but with no other symptoms commonly caused by pheochromocytoma. She developed massive bleeding and hemodynamic fluctuations during surgical removal of the tumor. A diagnosis of metastatic pheochromocytoma in the spine was made based on the results of intraoperative biopsy and postoperative examination. Both the patient and the fetus survived.

## Case presentation

A 33-year-old, 17-week pregnant woman (height 165 cm, weight 47.7 kg) visited the department of orthopedic surgery of a neighborhood hospital with a chief complaint of back pain. Medical history was unremarkable except ventricular extrasystoles on electrocardiogram since childhood. She was transferred to our hospital for further examination, with a suspicious diagnosis of a spinal tumor.

**Fig. 1:**
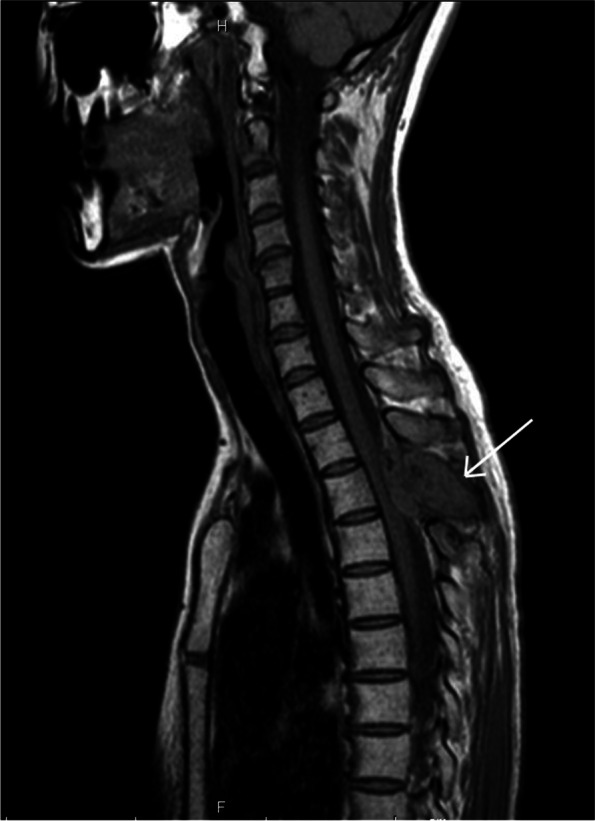
Preoperative sagittal T1-weighted magnetic resonance imaging revealed a mass in the posterior portion of the third thoracic vertebra

On admission, she was not able to walk unaided due to decreased muscle strength of bilateral lower limbs. Hypoesthesia below the seventh thoracic dermatome with increased bilateral patellar tendon and Achilles tendon reflex was observed. Magnetic resonance imaging (MRI) revealed a mass in the third thoracic vertebra, with T1-weighted signal intensity similar to and T2-weighted intensity slightly lower than the spinal cord (Fig. 1), suggesting bone giant cell tumor, granular cell sarcoma due to leukemia, malignant lymphoma, and osteosarcoma. Electrocardiography showed scattered ventricular extrasystoles as noted previously. Blood tests demonstrated mild anemia and increased inflammatory reaction. Catecholamines in the blood or urine were not measured. As the patient experienced rapid progression of leg paralysis, urgent thoracic posterior decompression with fusion and interval tumor resection was planned.

**Fig. 2:**
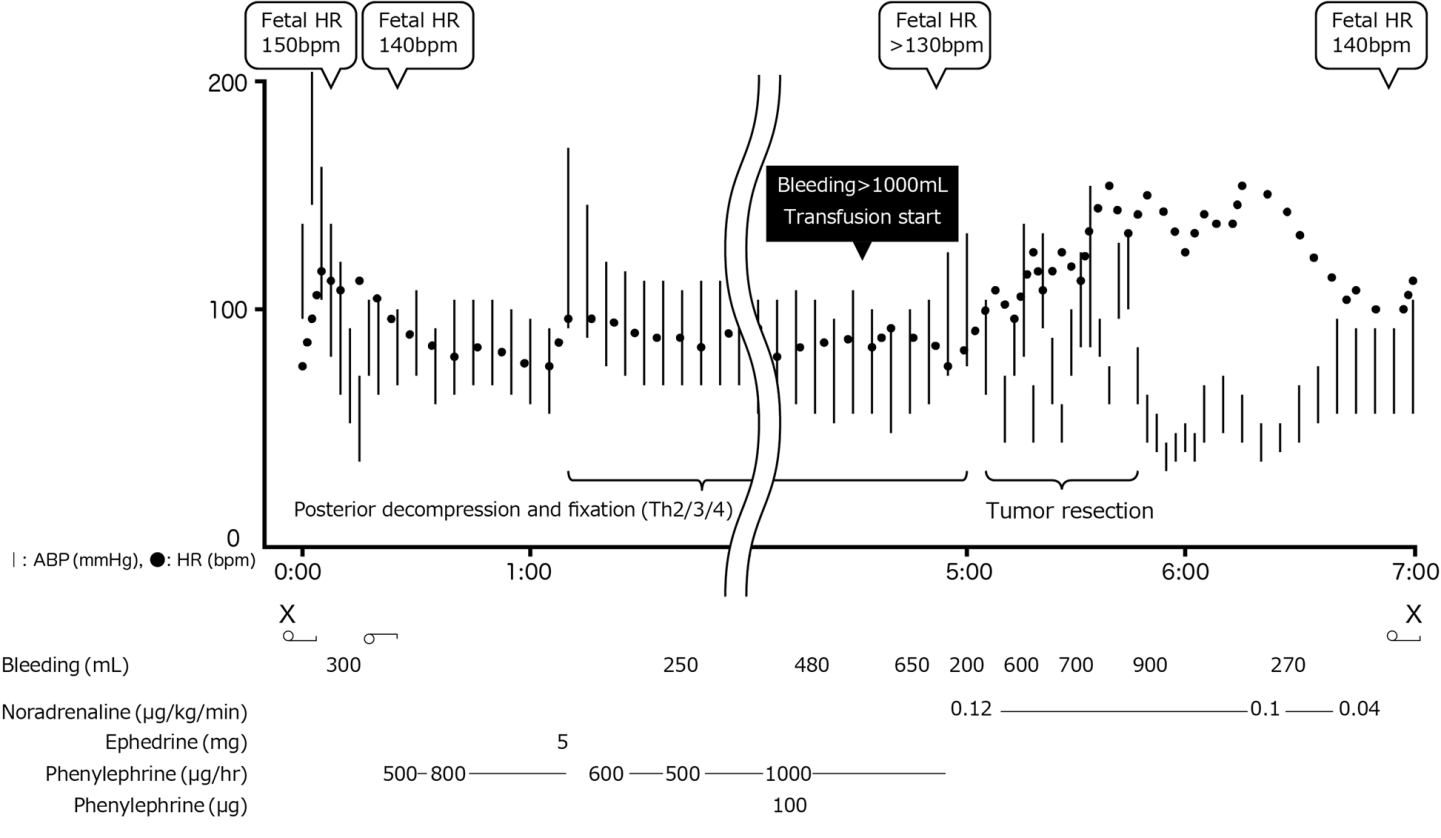
Anesthesia record. HR, heart rate; ABP, arterial blood pressure; X, anesthesia start/end

General anesthesia was induced with thiopental 200 mg, remifentanil 0.3 μg/kg/min, and rocuronium 40 mg followed by tracheal intubation and maintained with sevoflurane 1–1.5%, remifentanil 0.2–0.25 μg/kg/min, and bolus infusion of rocuronium 10 mg every 1 h. An abrupt increase of blood pressure to 223/162 mmHg with a heart rate of 103 bpm, followed by hypotension to 70/35 mmHg, was noted after tracheal intubation, but with small hemodynamic changes after changing the position to the prone. Fetal heart rate was intermittently monitored by an obstetrician using the ultrasound Doppler method to assess fetal well-being during the operation. Fetal heart rate was 150 bpm in the supine position and 140 bpm after the postural change to the prone position. When the bleeding exceeded 1000 mL, blood transfusion was started and fetal heart rate was confirmed to be > 130 bpm. Marked bleeding and hypertension (170/101 mmHg) were noted during tumor resection, immediately followed by hypotension (63/43 mmHg) during the suspension period of surgical resection despite massive blood transfusion (Fig. 2). Continuous infusion of noradrenaline was started. Resection of the tumor was abandoned, and only a biopsy was performed because of the persistent hypotension. Fetal heart rate was stable and maintained between 130 and 140 bpm. Intraoperative bleeding amounted to 4350 mL, and the total transfusion volume was 3930 mL (red blood cell concentrate, 16 units; fresh frozen plasma, 12 units; and platelet concentrate, 20 units). The operating time was 338 min. The patient was transferred to the intensive care unit (ICU) with continuous administration of noradrenaline, propofol, and tracheal intubation. Six hours after surgery, the patient was extubated in the ICU with the discontinuation of propofol.

On the following day, the circulatory dynamics improved, and noradrenaline was discontinued. The patient subsequently transferred to an inpatient orthopedic unit. Histopathological examination revealed that the tumor was a metastatic pheochromocytoma. Serum and urinary catecholamine levels were increased (serum adrenalin < 0.01 ng/mL, serum noradrenalin = 6.1 ng/mL, serum dopamine = 1.8 ng/mL, urinary adrenalin = 0.12 μg/day, urinary noradrenalin = 10 μg/day). Administration of doxazosin mesilate and carvedilol was started after the diagnosis. Fetal ultrasound on postoperative day 23 showed favorable fetal growth but indicated a tumorous lesion in the vesicouterine excavation. Based on the results of urgent pelvic MRI and needle biopsy, a definitive diagnosis of extra-adrenal pheochromocytoma in the urinary bladder with spinal metastasis was made. Since the continuation of pregnancy was life-threatening and posed a high risk to the patient, elective abortion was performed at 21 weeks and 4 days of pregnancy after discussion with the patient to prioritize the treatment of the underlying disease. Subsequently, the patient received chemotherapy and underwent surgeries for the residual tumor in the spine and the primary lesion, which led to remission.

## Discussion

We have presented a pregnant woman who experienced unexpected hypertension/hypotension and massive bleeding during resection of an undiagnosed spinal tumor. Surgery was discontinued after agreement with the surgeons for prioritizing the lives of the patient, fetus, and stabilizing their hemodynamics. Although the patient was diagnosed with metastatic pheochromocytoma on histopathological examination, it was not suspected preoperatively due to the absence of symptoms suggestive of it such as headache, nausea, palpitation, abnormal sweating, and hypertension. Preoperative spinal MRI was not specific as pheochromocytoma, because it has various MRI appearances [6]. Serum or urine catecholamine levels were not measured preoperatively; 123I-metaiodobenzylguanidine scintigraphy, specific for detecting pheochromocytoma, was a contraindication in a pregnant woman.

Only one case of metastatic pheochromocytoma to the spine has been reported during pregnancy [4]. Although her complaint was only numbness, decreased muscle strength, and urinary incontinence, abdominal CT and MRI revealed the primary tumor in the adrenal gland. In the present case, preoperative systolic blood pressure was slightly high (approximately 130 mmHg) for the patient’s age but was within the normal range. Combined with the primary lesion in the urinary bladder with these findings, it was difficult to arrive at a precise diagnosis preoperatively. Pheochromocytoma could have been suspected based on marked tachycardia and hypertension during tumor manipulation. In addition, a detailed interview after the diagnosis revealed that the patient had been aware of palpitations during bowel movements since she was in school, which is considered a symptom of pheochromocytoma.

The present case has many implications for the issue of fetal assessment in pregnant patients undergoing surgery. Clinical guidance from a committee of the American College of Obstetricians and Gynecologists recommends continuous intraoperative fetal heart rate monitoring only when the fetus is viable and an emergency cesarean delivery is possible [7]. In addition, previous case series of spine surgery on pregnant women have reported that continuous fetal heart rate monitoring is not generally performed in surgeries before the 20th week of gestation, because abnormal heart rate patterns cannot be assessed using continuous fetal heart rate monitoring until about 24 weeks of gestation [8]. Therefore, in the present case, we planned to monitor the fetal heart rate intermittently with ultrasound Doppler instead of continuous intraoperative fetal heart rate monitoring. However, in this case, the patient had an unexpected pheochromocytoma, which significantly altered the patient’s hemodynamics during surgery. As a result, it was difficult to monitor the fetal heart rate frequently during the operation because the anesthesiologist was busy stabilizing the maternal circulatory system, but the fact that we were able to confirm the survival of the fetus several times during the operation gave the anesthesiologist peace of mind. For atypical surgeries during pregnancy, it is recommended that arterial pressure monitoring and intermittent fetal heart rate monitoring should be prepared for unexpected situations.

Immediately after the operation, the maternal hemodynamics were considered to be unstable. Therefore, the patient was transferred to the ICU under sedation with propofol and extubated 6 h later. Considering the effect of propofol on the fetus, the amount and administration time of propofol should be kept to the minimum necessary in such cases. Preoperative discussion about the extent of resection of the tumor with the surgeons, implicating the malignancy of the tumor, would also be important.

## Conclusion

We have reported a case of a pregnant patient with spinal metastatic pheochromocytoma. The incidence of pregnancy complicated by a metastatic pheochromocytoma is extremely low. However, when acute drastic changes in circulatory dynamics are observed during resection of an undiagnosed spinal tumor in a pregnant patient, it is necessary to stabilize the maternal circulatory dynamics and to maintain the fetal blood flow, considering the possibility of metastatic pheochromocytoma.

## Data Availability

Not applicable.

## Abbreviations

MRI: Magnetic resonance imaging
T1WI: T1-weighted imaging
T2WI: T2-weighted imaging
ICU: Intensive care unit
